# P-1438. Health-Related Social Needs and Guardian Adversity in Outpatient Pediatric Infectious Diseases

**DOI:** 10.1093/ofid/ofae631.1612

**Published:** 2025-01-29

**Authors:** Melissa E Day, Chitravati Choony, Qing Duan, Mary Carol Burkhardt, Melissa Klein, Andrew F Beck, Elizabeth P Schlaudecker

**Affiliations:** Cincinnati Children's Hospital Medical Center, Cincinnati, Ohio; Cincinnati Children's Hospital Medical Center, Cincinnati, Ohio; Cincinnati Children's Hospital Medical Center, Cincinnati, Ohio; Cincinnati Children's Hospital Medical Center, Cincinnati, Ohio; Cincinnati Children's Hospital Medical Center, Cincinnati, Ohio; Cincinnati Children's Hospital Medical Center, Cincinnati, Ohio; Cincinnati Children's Hospital Medical Center, Cincinnati, Ohio

## Abstract

**Background:**

Infectious Diseases (ID) outpatient settings do not routinely screen for psychosocial needs of patients and families that may exacerbate infectious processes or challenge traditional treatment. We assessed the prevalence of health-related social needs (HRSN), guardian adverse childhood experiences (ACEs), and neighborhood-level socioeconomic deprivation in two pediatric outpatient ID clinics.

**Figure:**
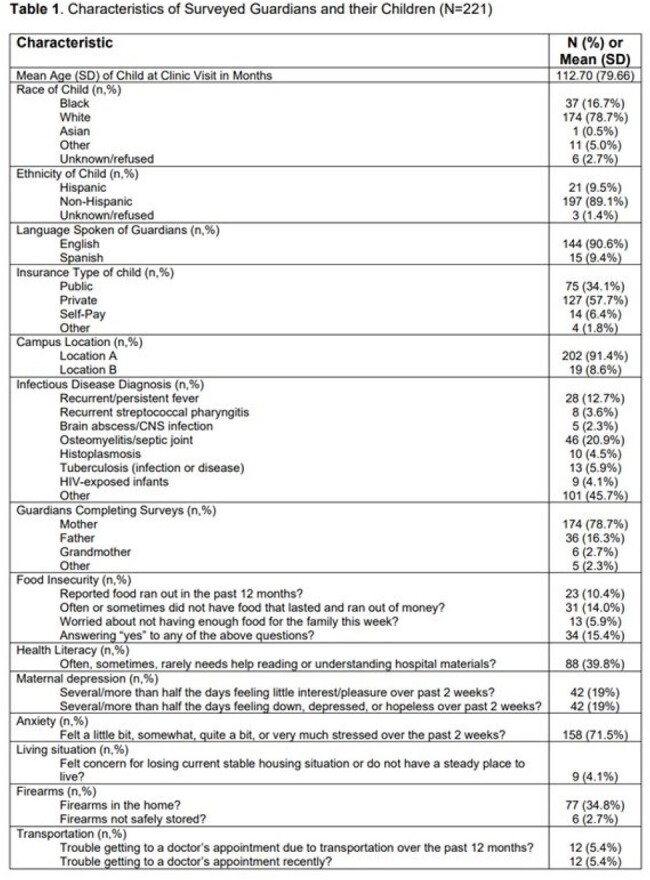

**Methods:**

Using a cross-sectional study design, we approached English- and Spanish-speaking guardians of outpatient ID patients at two campuses. Guardians completed a HRSN screen (evaluating food/housing insecurity, transportation access, and mental health concerns) and the 10-question ACEs questionnaire (evaluating traumatic childhood experiences of guardians). Responses were entered into REDCap. We geocoded street addresses to identify census tract-level socioeconomic deprivation index (SDI) scores, with higher scores indicating more deprivation. We quantified individual HRSN items, total ACE scores, and SDI measures. We extracted clinical/demographic information from patient charts. Descriptive analyses were pursued. ANOVA analyses explored associations with psychosocial needs and SDI scores.

**Figure 1.**
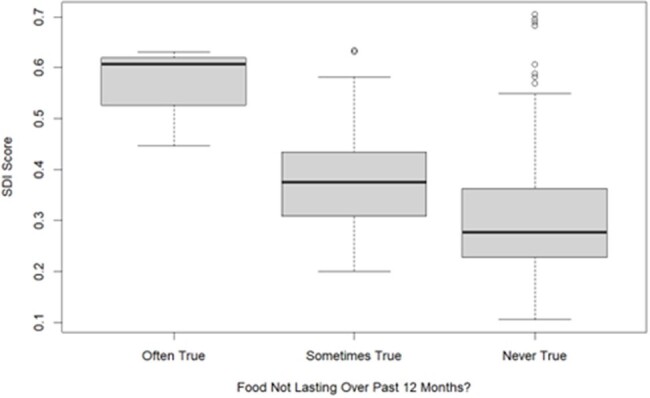
Association of SDI score and Food Insecurity Over the Past 12 Months.

**Results:**

To date, 221 caregivers have been surveyed, and results are described in **Table 1**. Most children identified as White, non-Hispanic, and English-speaking. Many families reported any food insecurity (15%), regular caregiver depressive symptoms (20%), and recent anxiety (70%). Four percent lived in an unsteady place, and 5% had transportation issues that affected access to medical care. Firearms were present in 35% of homes. Nineteen percent of guardians had high ACEs (≥4). The mean SDI score was 0.32 (range 0.11-0.71), similar to our institution’s primary service area. Higher SDI scores were associated with more food insecurity over the past 12 months (**Figure 1**, p< 0.001) and the past week (**Figure 2**, p=0.01).

**Figure 2.**
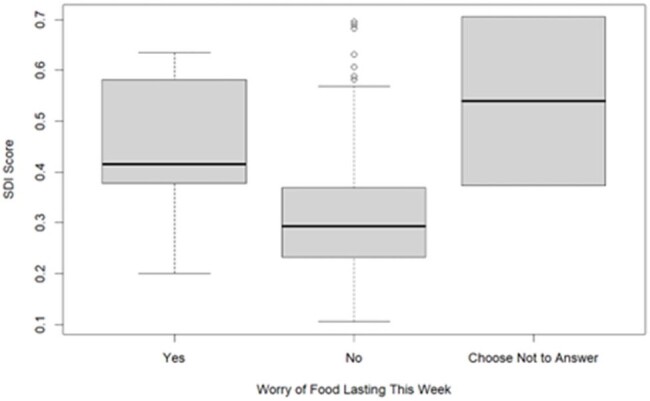
Association of SDI Score and Food Insecurity Over the Past Week.

**Conclusion:**

Guardians of children receiving ID outpatient care reported HRSNs and ACEs. SDI score may be a useful adjuvant to screening to identify more efficiently those at risk for challenges like food insecurity. Outpatient ID settings can identify and help mitigate these concerns in clinical encounters.

**Disclosures:**

**Elizabeth P. Schlaudecker, MD, MPH**, Pfizer: Grant/Research Support|Sanofi Pasteur: Advisor/Consultant

